# Hippocampal volume in patients with bilateral and unilateral peripheral vestibular dysfunction

**DOI:** 10.1016/j.nicl.2022.103212

**Published:** 2022-09-28

**Authors:** Corina G. Schöne, Michael Rebsamen, Gerda Wyssen, Christian Rummel, Franca Wagner, Dominique Vibert, Fred W. Mast

**Affiliations:** a Department of Psychology, University of Bern, Bern, Switzerland; b Department of Otorhinolaryngology, Head and Neck Surgery, Inselspital, University Hospital Bern, University of Bern, Bern, Switzerland; c Doctoral Program for Brain and Behavioral Sciences, University of Bern, Bern, Switzerland; d Support Center for Advanced Neuroimaging (SCAN), University Institute of Diagnostic and Interventional Neuroradiology, Inselspital, University Hospital Bern, University of Bern, Bern, Switzerland; e Graduate School for Cellular and Biomedical Sciences, University of Bern, Bern, Switzerland

**Keywords:** Vestibular dysfunction, Brain morphometry, MRI, Hippocampus, Presubiculum, Supramarginal gyrus, PVD, peripheral vestibular dysfunction

## Abstract

Previous studies have found that peripheral vestibular dysfunction is associated with altered volumes in different brain structures, especially in the hippocampus. However, published evidence is conflicting. Based on previous findings, we compared hippocampal volume, as well as supramarginal, superior temporal, and postcentral gyrus in a sample of 55 patients with different conditions of peripheral vestibular dysfunction (bilateral, chronic unilateral, acute unilateral) to 39 age- and sex-matched healthy controls. In addition, we explored deviations in gray-matter volumes in hippocampal subfields. We also analysed correlations between morphometric data and visuo-spatial performance. Patients with vestibular dysfunction did not differ in total hippocampal volume from healthy controls. However, a reduced volume in the right presubiculum of the hippocampus and the left supramarginal gyrus was observed in patients with chronic and acute unilateral vestibular dysfunction, but not in patients with bilateral vestibular dysfunction. No association of altered volumes with visuo-spatial performance was found. An asymmetric vestibular input due to unilateral vestibular dysfunction might lead to reduced central brain volumes that are involved in vestibular processing.

## Highlights

• Morphometric analysis of 55 patients with peripheral vestibular dysfunction.
• No hippocampal volume reduction compared to healthy controls.
• Reduced right presubiculum in patients with unilateral vestibular dysfunction.
• Reduced left supramarginal gyrus in patients with unilateral vestibular dysfunction.

## 1 Introduction

Peripheral vestibular dysfunction (PVD) describes missing or reduced vestibular function due to a disease of the inner ear vestibular structures or the vestibular nerve (Herdman & Clendaniel, 2014). PVD can appear in various conditions: 1) laterality: The dysfunction can affect one side (unilateral PVD) or both sides (bilateral PVD). 2) course: Patients with PVD can recover from initial PVD and regain a normal peripheral vestibular function (acute PVD), especially when rehabilitation starts early after PVD (Lacour, 2006). However, some patients develop a chronic state with persistent absence or reduction of vestibular function (chronic PVD). Research including patients with PVD is often limited to only one condition of PVD (unilateral PVD/bilateral PVD/acute PVD/chronic PVD).

Various morphometry studies observed altered brain structure in patients with different conditions of PVD (bilateral/chronic unilateral/acute unilateral) compared to healthy controls, predominantly in the hippocampus (Brandt et al., 2005, Göttlich et al., 2016, Hong et al., 2014, Kremmyda et al., 2016, Seo et al., 2016, zu Eulenburg et al., 2010). There are also conflicting results showing no altered hippocampal volume in patients with different conditions of PVD (bilateral/chronic unilateral/acute unilateral) (Cutfield et al., 2014, Helmchen et al., 2009, Helmchen et al., 2010, Hüfner et al., 2007, Hüfner et al., 2009, van Cruijsen et al., 2007). In patients with chronic unilateral PVD Hüfner et al. (2007) did not find a reduced hippocampal volume, however, patients had a smaller volume in the postcentral, superior temporal and supramarginal gyrus (Hüfner et al., 2009). A reduced volume of the superior temporal gyrus was not only found in patients with chronic unilateral PVD (Helmchen et al., 2010, Hüfner et al., 2009), but also in patients with acute unilateral PVD (Helmchen et al., 2009).

The hippocampus plays a central role in visuo-spatial cognitive performance and patients with PVD have been shown to perform worse in visuo-spatial tasks than healthy controls (Gammeri et al., 2022, Hanes and McCollum, 2006). However, knowledge about correlations between morphometric data and behavioral performance is limited. A study by Brandt et al. (2005) reported that deficits in visuo-spatial performance correlated with hippocampal atrophy in patients with PVD, while other studies found no such association (Hüfner et al., 2009, Kremmyda et al., 2016). Other morphometry studies in patients with PVD did not include spatial performance tests.

In this study, we analysed brain structures from prospectively acquired MRI in a large sample of 55 patients with different conditions of PVD (bilateral, chronic unilateral, acute unilateral) and 39 age- and sex-matched healthy controls. Rather than exploratively investigating the entire brain, we concentrated on regions of interest that were reported as abnormal in the literature. While previous studies primarily compared total hippocampal volumes, we extended this line of research by also exploring the hippocampal subfields. In addition, we compared gray-matter changes in supramarginal, superior temporal, and postcentral gyrus between patients with PVD and healthy controls to reproduce the findings of Hüfner et al. (2009) on this prospectively acquired dataset. Furthermore, we correlated morphometric data with performance in a visuo-spatial task (mental body rotation) to investigate whether altered volumes correlate with visuo-spatial performance.

## 2 Materials and methods

### 2.1 Ethical considerations

The study was conducted in agreement with the Declaration of Helsinki. The study protocol was approved by the ethics committee of the Canton Bern, Switzerland. All participants gave their written informed consent prior to study participation.

### 2.2 Participants

#### 2.2.1 Patients

Patients were recruited from the Department of Otorhinolaryngology, Head and Neck Surgery, of the University Hospital of Bern, Switzerland. Initially, 65 patients with a diagnosis of PVD participated in this study. Chronic PVD was defined by a PVD of at least 6 months after diagnosis, whereas acute PVD was defined by a PVD of maximum one month after diagnosis (Brandt, 1999, Strupp et al., 2020). The lesion side in patients with unilateral PVD was not balanced in the recruitment process. Diagnosis of PVD was based on the results of neurotological examination, interpreted by an experienced neurotologist (D. Vibert). The neurotological examination included video-electronystagmography (VNG) with bithermal caloric testing, video head impulse test (V-HIT), cervical vestibular evoked myogenic potentials (cVEMPs), ocular vestibular evoked myogenic potentials (oVEMPs) and dynamic posturography (SwayStar). The degree of vestibular deafferentation was defined as complete in case of an absence of nystagmic response or incomplete in case of a unilateral weakness of > 20 % of the lateral semicircular canal in caloric testing. Subjective vertigo-related disability was assessed with a German version of the dizziness handicap inventory (DHI-G, Kurre et al., 2009). Patients were excluded from the study if they fulfilled one of the following criteria: central vestibular dysfunction (defined as the presence of vertigo or dizziness associated with or without neurological signs, whereas neurotological examination was normal); serious cardiovascular, metabolic, neurologic or degenerative disease; neuroleptics; cerebral concussion during the year prior to the study; contraindications for MRI assessment (e.g. pregnancy, pacemaker, cochlear implant) or insufficient German language skills.

#### 2.2.2 Healthy controls

As a control group, 46 healthy volunteers were recruited by newspaper advertisements and word of mouth. Participants were matched regarding age, sex, handedness, and education to patients with PVD. All participants underwent a neurotological examination to exclude vestibular dysfunction. The neurotological assessment included the same tests as for patients. Exclusion criteria for healthy participants were the same as in patients (described above).

### 2.3 Materials and study procedure

#### 2.3.1 MRI acquisition

The visits for MRI acquisition lasted approximately one hour. For morphometry, high-resolution, T1-weighted MR images were acquired on a 3 T scanner (Siemens Magnetom Prisma, Siemens, Erlangen, Germany) using the MP-RAGE protocol (van der Kouwe et al., 2008), (TI = 1100 ms, TR = 2330 ms, TE = 3.03 ms, flip angle = 8°, 1 mm isotropic resolution). A Siemens Head/Neck 64-channel coil was used. During the body rotation task, participants answered by response buttons with the left or the right index finger.

#### 2.3.2 Mental body rotation task

Visuo-spatial performance of participants was measured by means of a mental body rotation task previously used in patients with PVD (Grabherr et al., 2011). This task is influenced by concurrent caloric vestibular stimulation (Klaus et al., 2020) and visually simulated self-motion has been shown to activate vestibular associated brain areas such as the hippocampus (Indovina et al., 2013). Participants were presented with a picture of a person that stretched out either the left or the right arm. This picture was presented in different orientations. Participants had to imagine rotating their own body until they adopted the same orientation as the person on the screen. After the mental rotation they indicated by button press whether the person on the screen stretched out the left or the right arm. We assessed accuracy (correct, incorrect) and response times.

### 2.4 Data analysis

#### 2.4.1 Morphometric analysis

The images were processed with FreeSurfer 6.0 (Fischl, 2012) to extract volumes of subcortical (Fischl et al., 2002) and cortical (Dale et al., 1999) regions of interest (ROI). Regional volumes were corrected for normalized (zero-mean, unit SD) age and estimated total intracranial volume (eTIV) as a measure of brain size (Buckner et al., 2004) by fitting a linear model on the healthy controls and subsequent application of the coefficients on all subjects. The covariate sex was omitted as not directly related to the volumes (Im et al., 2008, Jäncke et al., 2015). Group-wise comparisons were conducted with these corrected volumes using two-sided t-tests. For patients with unilateral PVD, we also compared (uncorrected) volumes of the ipsilateral (side of the vestibular dysfunction) to the contralateral side using paired t-tests. Statistical analyses were performed in *R* (version 3.6.2, R Core Team, 2019) using a significance level of α = 0.05.

Besides the hypothesis-driven analysis of selected ROI, we explored further traits of the hippocampus that might be characteristic for the disease: hippocampal subfields and the shape of the hippocampus. Hippocampal subfields were segmented using a dedicated module (Iglesias et al., 2015) of the FreeSurfer pipeline. Additionally, the images were processed using *DL + DiReCT* (Rebsamen et al., 2020, Rebsamen et al., 2022), from which 16 shape features of the hippocampi were derived using *pyradiomics* (van Griethuysen et al., 2017). With a data-driven approach, we investigated the discriminatory power of these features to classify patients with vestibular dysfunction and its subgroup from healthy controls using a machine-learning classifier. Three distinct subsets of features were used as input (Supplementary Table S1): Volumes of anatomical ROIs from FreeSurfer (n = 28 features), radiomics shape features of the hippocampi (n = 28), and volumes of hippocampal subfields (n = 26). For each feature set, a linear support vector machine (SVM) (Chang and Lin, 2011, Pedregosa et al., 2011) was trained using fivefold cross-validation and 20 repeats. Performance was evaluated by calculating the area under the receiver operating characteristic curve (AUC) averaged over all folds. The most discriminatory feature based on relative feature importance was further analysed with statistical tests and boxplots.

To exclude influence of demographic confounders on results, we repeated our analysis with a 1:1 matching. For each patient we selected a healthy control participant out of our sample comparable to the patient in age, sex, handedness and education. As these analyses did not differ from the group analysis, we do not report the analysis from the 1:1 matching.

#### 2.4.2 Correlations of morphometric data with visuo-spatial task performance

Regions of altered brain volumes, specifically the volume of the right presubiculum and the left supramarginal gyrus were correlated with the response times and accuracy in the body rotation task. In addition, we investigated whether a processing speed task, that was conducted for another study (Color-Word Interference test, D-KEFS, Delis et al., 2001), correlated with the volume of the right presubiculum. Data of 37 controls and 52 patients (18 bilateral PVD, 21 chronic unilateral PVD, 13 acute unilateral PVD) was included. We excluded data of participants who did not solve the task (n = 3), or solved it with an accuracy below 50 % in the control condition (n = 2). Bayesian Kendall’s Tau correlation coefficients were calculated due to not normally distributed variables (i.e. accuracy) and possible nonlinear relationships (e.g. exponential). Correlation coefficients and 95 % credible intervals were calculated in JASP (version 0.16.1, JASP Team, 2022) using default priors (stretched beta prior width = 1). Bayes factors were used to estimate if the data was more likely under the two-sided alternative than under the null hypothesis.

## 3 Results

### 3.1 Data exclusion

From initially 65 patients, ten patients were excluded due to incidental findings in MRI (n = 2), recovered vestibular function (n = 5), exclusion criteria that the patients did not disclose prior to study participation (n = 2), or movement artefacts (n = 1). Therefore, we analysed data from 55 patients (see Table 1 for demographic and clinical data). Patients either had bilateral PVD (n = 19), chronic unilateral PVD (n = 21; right-sided lesion n = 9, left-sided lesion n = 12) or acute unilateral PVD (n = 15; right-sided lesion n = 10, left-sided lesion n = 5). The most common diagnosis was idiopathic in patients with bilateral PVD (10 out of 19), vestibular schwannoma in patients with chronic unilateral PVD (14 out of 21) and vestibular neuritis in patients with acute PVD (14 out of 15). Detailed diagnosis and lesion side of each patient are shown in Supplementary Table S2.

**Table 1:** Demographic and clinical data of the patient groups with different conditions of peripheral vestibular dysfunction (bilateral, chronic unilateral, acute unilateral) and healthy controls.

	Bilateral PVD (n = 19)	Chronic unilateral PVD (n = 21)	Acute unilateral PVD (n = 15)	Healthy controls (n = 39)
Sex (female/male)	5/14	6/15	5/10	17/22
Age (years) a	53.18 ± 19.00	56.52 ± 11.08	47.35 ± 15.77	52.07 ± 16.53
Handedness (right/left)	17/2	18/3	11/4	32/7
Disease duration a	14.68 ± 13.29 years	8.66 ± 6.03 years	21.47 ± 3.58 days	–
Lesion side (right/left)	–	10/11	10/5	–
Degree of vestibular deafferentation b (incomplete/complete)	7/12	10/11	12/3	–
DHI-G score a	25.03 ± 17.91	27.95 ± 19.66	35.53 ± 27.42	–

*Note. DHI-G* score. German version of the Dizziness Handicap Inventory.

a Results are presented as mean ± standard deviation.

b Degree of vestibular deafferentation was measured with bithermal caloric testing.

From initially 46 healthy controls, seven controls were excluded due to incidental findings in MRI (n = 3), exclusion criteria that the participants did not disclose prior to study participation (n = 2), or extremely low performance (≤ 3 SD / ≤ 2 SD) of global scores of cognitive functions and intelligence that were assessed for another study (n = 2). Therefore, data from 39 healthy controls were analysed (see Table 1 for demographic data).

### 3.2 Morphometric analysis

No statistically significant differences of total hippocampal volumes were observed, neither between healthy controls (mean corrected volume left hippocampus = 3.950 ml, right = 4.099 ml) and all patients with PVD (left = 4.010 ml; p = 0.43, right = 4.151 ml; p = 0.51), nor between the subgroups of bilateral (left = 3.999 ml; p = 0.65, right = 4.144 ml; p = 0.68), acute unilateral (left = 4.001 ml; p = 0.6, right = 4.203 ml; p = 0.36) or chronic unilateral (left = 4.026 ml; p = 0.45, right = 4.121 ml; p = 0.84) and matched healthy controls. A comparison of these observations to results from previous studies is shown in Table 2.

**Table 2:** Comparison of previous brain morphometry studies on vestibular dysfunction and results in this study.

Study	Number of patients	Number of healthy controls	Method	Main Finding Hippocampus	Agreement with our Findings
Brandt et al., 2005	10	10	Manual	Volume loss	No
Göttlich et al., 2016	27	29	VBM SPM12	No change	Yes
Kremmyda et al., 2016	15	15	VBM SPM8	Volume loss mid-hippocampus	–
Cutfield et al., 2014	12	15	VBM FSL	No change	Yes
van Cruijsen et al., 2007	10	10	Manual	No change	Yes
zu Eulenburg et al., 2010	22	22	VBM SPM5	Decreased intensities left posterior hippocampus	–
Seo et al., 2016	38	76	Manual	Volume loss	No
Hüfner et al., 2007	16	16	Manual / VBM SPM2^1^	No change	Yes
Hüfner et al., 2009	16	16	VBM SPM2	No change	Yes
Helmchen et al., 2009	15	15	VBM SPM2	No change	Yes
Helmchen et al., 2010	15	15	VBM SPM2	No change	Yes
Hong et al., 2014	9	–	VBM SPM8	−^2^	–
**This Study**	55	39	FreeSurfer 6		

^1^ Manual tracing of hippocampus / SPM2 for cortex.

^2^ Longitudinal study, no comparison to healthy control group.

Likewise, no volume differences were found for the left and right postcentral and superior temporal gyrus (Supplementary Fig. S1). However, we observed a reduced cortical gray-matter volume in the left supramarginal gyrus in the subgroups of patients with acute unilateral (p = 0.018) and chronic unilateral (p = 0.011) PVD as depicted in Supplementary Fig. S2. This difference was present independent of the laterality of the disease.

No statistically significant difference was found comparing the volumes of the ipsi- to the contralateral side, neither for the hippocampus (p = 0.24) nor the postcentral (p = 0.8), superior temporal (p = 0.69) or supramarginal gyrus (p = 0.94).

**Fig. 1:**
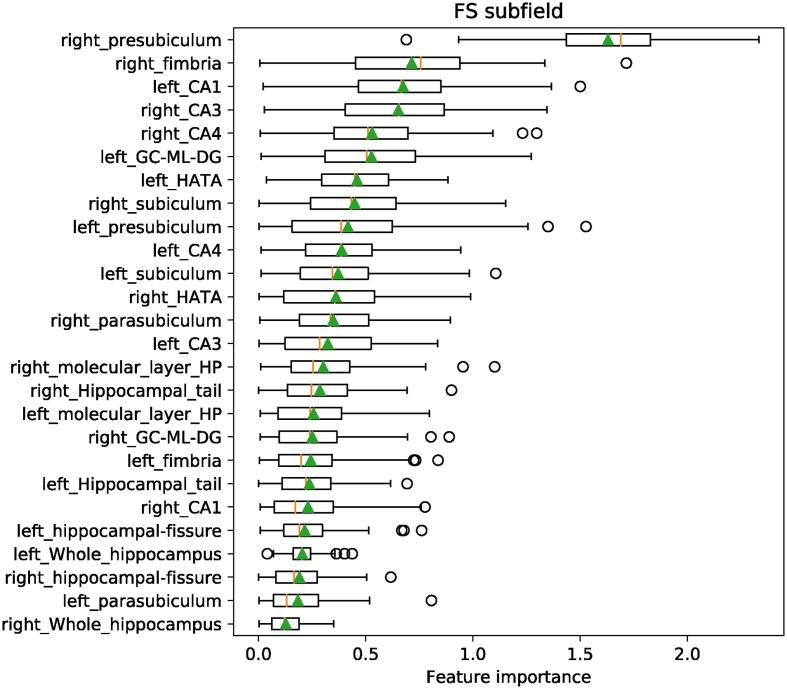
Relative feature importance of the classifier using the hippocampal subfields to separate patients with PVD from healthy controls.

The classifier using the hippocampal subfields as features performed slightly better than chance for the entire cohort (AUC = 0.565) and substantially better for the subgroup of acute unilateral patients (AUC = 0.646) as shown in Supplementary Fig. S3. The volume of the right presubiculum was identified as the most discriminative feature (Fig. 1).

**Fig. 2:**
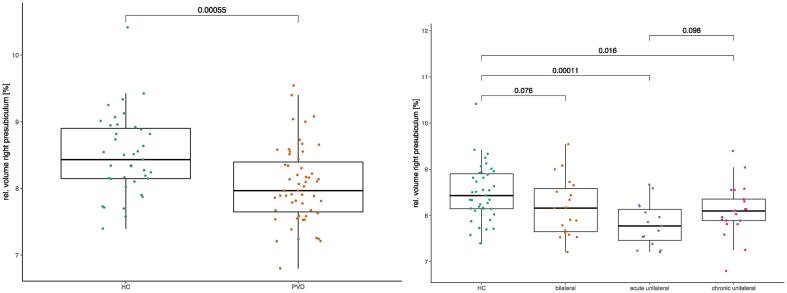
Boxplots of the relative volume of the right presubiculum comparing healthy controls (HC) to patients with peripheral vestibular dysfunction PVD (left) and to subgroups of bilateral, acute unilateral and chronic unilateral PVD (right). P-values from two-sided t-tests.

**Fig. 3:**
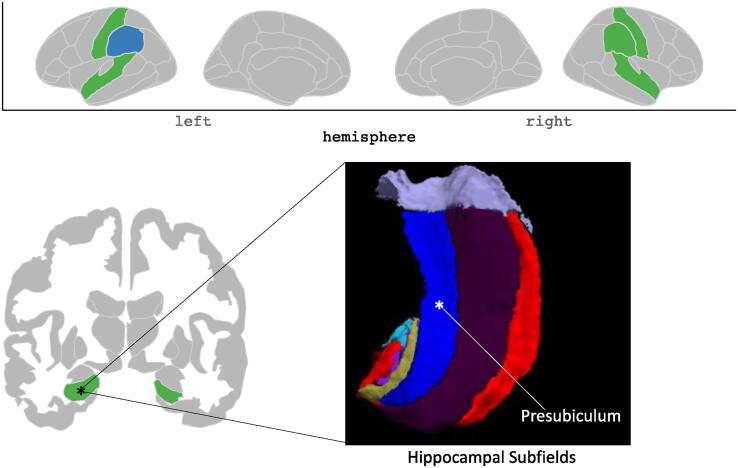
Graphical summary of the results. Investigated regions are highlighted in green (hippocampus, supramarginal gyrus, superior temporal gyrus and postcentral gyrus). Statistically significant reduced volumes of patients with unilateral vestibular dysfunction compared to healthy controls are highlighted in blue (left supramarginal gyrus and right presubiculum). The hippocampal subfields of the right hippocampus (in radiological orientation) are shown inferior-superior. (For interpretation of the references to color in this figure legend, the reader is referred to the web version of this article.)

Indeed, the volume of the right presubiculum relative to the whole hippocampus was reduced in patients with PVD (mean volume fraction = 8.1 %, p < 10^−3^) compared to healthy controls (8.5 %), induced by the subgroup of acute unilateral (7.8 %, p = 0.0001) and chronic unilateral patients (8.1 %, p = 0.016) as depicted in Fig. 2. The effect was present independent of the laterality of the disease (Supplementary Fig. S4). No volume reduction was observed for the left presubiculum (Supplementary Fig. S5). A summary of the analysed and statistically significant regions is shown in Fig. 3.

### 3.3 Correlations of morphometric data with visuo-spatial task performance

For all calculated correlations, 95 % credible intervals included zero, and Bayes factors BF_10_ were between 0.1 and 3, showing neither evidence for a correlation nor for the absence of a correlation between the visuo-spatial performance measures and the right presubiculum and left supramarginal gyrus volumes (see Supplementary Tables S3-S4 and Supplementary Figs. S6-S7).

## 4 Discussion

We have analysed structural changes in MRI of the brain in a large sample of 55 patients with PVD compared to 39 healthy controls matched for age, sex, handedness, and education. In agreement with several previous studies (cf. Table 2), we have observed no significant difference in total hippocampal volume, neither in the entire patient cohort nor in the different conditions of PVD (bilateral, chronic unilateral, acute unilateral). Interestingly, patients with unilateral PVD (chronic and acute) had a smaller volume in the right presubiculum than healthy controls, but not patients with a bilateral PVD. Replicating findings from a previous study (Hüfner et al., 2009), we found a reduced cortical volume of the left supramarginal gyrus in patients with unilateral PVD (chronic and acute), but not in patients with bilateral PVD. In contradiction to the same study, the patient cohort tested in this study did not show an altered volume in the postcentral gyrus. Reduced volumes in the right presubiculum and the left supramarginal gyrus in patients with unilateral PVD did not correlate either with visuo-spatial performance nor the side of the lesion.

### 4.1 No reduced volume in total hippocampus, postcentral gyrus, and superior temporal gyrus

Normal hippocampal volumes in different conditions of PVD confirm some results of previous studies (Cutfield et al., 2014, Göttlich et al., 2016, Helmchen et al., 2009, Helmchen et al., 2010, Hüfner et al., 2007, Hüfner et al., 2009, van Cruijsen et al., 2007), see Table 2. However, it is in conflict with studies that report a smaller hippocampal volume in patients with bilateral PVD (Brandt et al., 2005) and chronic unilateral PVD (Seo et al., 2016). One possible reason for diverging results could be different degrees of vestibular deafferentation. In the study of Brandt et al. (2005) all patients had undergone a bilateral vestibular neurectomy and therefore suffered from complete vestibular deafferentation, whereas in our study seven out of 19 bilateral patients had an incomplete deafferentation of the lateral semicircular canal. Seo et al. (2016) did not differentiate between complete or incomplete deafferentation in their sample of chronic unilateral patients. In our study, ten out of 21 chronic unilateral patients suffered from incomplete deafferentation of the lateral semicircular canal. Some researchers suggest that hippocampal atrophy and spatial navigation deficits occur less frequently in patients with incomplete vestibular deafferentation (Göttlich et al., 2016).

In contrast to the study of Hüfner et al. (2009), we did not observe reduced volume in the postcentral and superior temporal gyrus in patients with PVD. All patients with vestibular schwannoma included in the study of Hüfner et al. (2009) had undergone schwannoma extirpation, compared to only five out of 14 patients in our study. Schwannoma extirpation might impair forwarding vestibular input, which in turn could affect volume in the postcentral gyrus.

### 4.2 Reduced volume in the right presubiculum and left supramarginal gyrus

A reduced volume in the right presubiculum in patients with unilateral PVD is a new finding, as no previous study investigated hippocampal subfields in patients with PVD. This reduction might be associated with the vestibular function of the presubiculum, which is involved in head direction coding (Finkelstein et al., 2015, Hitier et al., 2014, Robertson et al., 1999, Simonnet and Fricker, 2018, Taube, 2007, Wiener and Taube, 2005). A solely right-sided effect could be due to the lateralization of vestibular function in the right hemisphere (Dieterich & Brandt, 2018).

In agreement with findings from Hüfner et al. (2009), we observed reduced volume in the supramarginal gyrus in patients with unilateral PVD. However, contrary to Hüfner et al. (2009), we observed a reduced supramarginal gyrus on the left side and not ipsilateral to the lesion side. Interestingly, a study with healthy participants showed that processing of vestibular information and a mental body rotation task shared neural correlates in the left supramarginal gyrus (Klaus et al., 2020). Therefore, this region might be important for integrating vestibular information and the body’s spatial reference frame. However, as an assumption about the lateralization of altered brain areas was not the major aim of the study, there was a restricted sample size in each category (left/right). Therefore, we cannot make a concluding statement about the lateralization of altered brain areas.

### 4.3 Correlation of morphometric and behavioral data

Reduced volumes in the right presubiculum and the left supramarginal gyrus in patients with unilateral PVD did not correlate with visuo-spatial performance. This result is in line with a previous finding showing no correlation of the supramarginal gyrus with navigational abilities in patients with chronic unilateral PVD (Hüfner et al., 2009). However, Bayesian analysis did not provide evidence for the absence of correlations between morphometric and behavioural data. The analyses show an inconclusiveness, meaning it can’t be concluded that there is no correlation of morphometric data with visuo-spatial task performance. Future studies should investigate whether there is indeed no correlation. Other methodologies such as MR-based connectivity analysis or functional MRI may be more suitable to investigate the relationship between altered brain structures and visuo-spatial performance.

### 4.4 No reduced volumes in bilateral patients

Contrary to patients with unilateral PVD, patients with bilateral PVD did not have reduced volumes in the presubiculum or the supramarginal gyrus. This finding is surprising because both subgroups were comparable in size, and patients with bilateral PVD are expected to have more severe impairments. We speculate that the effect was not observed in patients with bilateral PVD for two reasons: First, patients with bilateral PVD in our study might have well adapted to their bilateral vestibular dysfunction. Indeed, patients with bilateral PVD had, on average, longer disease durations and lower scores in the Dizziness Handicap Inventory than patients with unilateral PVD (see Table 1). Second, contrary to patients with bilateral PVD, patients with unilateral PVD suffer from an asymmetrical vestibular input that might alter brain structures as an attempt to adapt to the asymmetric deafferentation.

### 4.5 Strengths and limitations

One important strength of our study is that we included groups with different conditions of PVD (laterality, course). High-resolution structural MRIs were acquired prospectively with identical protocols on the same scanner. Therefore, we could directly compare brain structures between different conditions of PVD. In addition, this is one of the largest samples of patients with PVD in a structural brain morphometry study on vestibular dysfunction. Furthermore, we included a healthy control group comparable to the patient group regarding age, sex, handedness, and education. Finally, we extended the line of research on the impact of PVD on hippocampal volume by further investigating anatomically defined subregions (hippocampal subfields).

Besides its strengths, our study has two limitations. First, although we included patients with different conditions of PVD (bilateral, chronic unilateral, acute unilateral), patients within the groups had heterogeneous diagnoses. However, the dysfunction of vestibular input is likely more influential on altered brain structure than the underlying specific diagnosis. Future studies will need to compare different conditions (laterality, course) and different diagnosis of PVD. Second, our observation of reduced volume in the presubiculum was identified in a data-driven, explorative fashion and remains to be confirmed in an independent cohort.

Comparisons to some of the previous studies is limited due to longitudinal nature of the study (Hong et al., 2014) or findings related to sub-regions of the hippocampus only (Kremmyda et al., 2016, zu Eulenburg et al., 2010). A direct comparison of other cortical regions identified by previous studies remains challenging due to the different methods applied (see Table 2), although surface-based morphometry is generally assumed to yield robust results and FreeSurfer the most widely used tool for that purpose (Clarkson et al., 2011).

## 5 Conclusion

Patients with different conditions of peripheral vestibular dysfunction (bilateral, chronic unilateral, acute unilateral) had no reduced total hippocampal volume compared to age- and sex-matched healthy controls. Contrary to bilateral vestibular dysfunction, chronic and acute unilateral vestibular dysfunction were associated with reduced brain volumes in the right presubiculum and the left supramarginal gyrus. Unilateral peripheral vestibular dysfunction might lead to reduced central brain volumes that are involved in the processing of vestibular information.

## CRediT authorship contribution statement

**Corina G. Schöne:** Conceptualization, Methodology, Software, Validation, Investigation, Project administration, Data curation, Writing – original draft, Writing – review & editing. **Michael Rebsamen:** Validation, Formal analysis, Data curation, Visualization, Writing – original draft, Writing – review & editing. **Gerda Wyssen:** Data curation, Formal analysis, Writing – original draft, Writing – review & editing. **Christian Rummel:** Supervision, Writing – review & editing. **Franca Wagner:** Supervision, Writing – review & editing. **Dominique Vibert:** Conceptualization, Resources, Writing – review & editing. **Fred W. Mast:** Conceptualization, Supervision, Funding acquisition, Writing – review & editing.

## Declaration of Competing Interest

The authors declare that they have no known competing financial interests or personal relationships that could have appeared to influence the work reported in this paper.

## Data Availability

The data that has been used is confidential.
