# Supplementary material for: Hippocampal volume in patients with bilateral and unilateral peripheral vestibular dysfunction
Source: Neuroimage Clin. 2022 Sep 28;36:103212. doi: 10.1016/j.nicl.2022.103212 (PMC9668627; doi:10.1016/j.nicl.2022.103212)
Supplement: Supplementary material 1 [file mmc1.pdf]

Supplementary Material

Supplementary Table S1: List of features used for the three classifiers. L: left, R: right.

FreeSurfer aseg	DL shape radiomics	Hippocampal Subfields
L/R VentralDC	L/R VoxelVolume	L/R subiculum
L/R Thalamus-Proper	L/R SurfaceVolumeRatio	L/R presubiculum
L/R Putamen	L/R SurfaceArea	L/R parasubiculum
L/R Pallidum	L/R Sphericity	L/R molecular-layer-HP
L/R Lateral-Ventricle	L/R MinorAxisLength	L/R hippocampal-fissure
L/R Inf-Lat-Vent	L/R MeshVolume	L/R fimbria
L/R Hippocampus	L/R Maximum3DDiameter	L/R Whole-hippocampus
L/R Cerebellum-White-Matter	L/R Maximum2DDiameterSlice	L/R Hippocampal-tail
L/R Cerebellum-Cortex	L/R Maximum2DDiameterRow	L/R HATA
L/R Caudate	L/R Maximum2DDiameterColumn	L/R GC-ML-DG
L/R Amygdala	L/R MajorAxisLength	L/R CA4
L/R Accumbens-area	L/R LeastAxisLength	L/R CA3
CortexVol	L/R Flatness	L/R CA1
CerebralWhiteMatterVol	L/R Elongation	
4th-Ventricle		
3rd-Ventricle		

Supplementary Table S2: Detailed medical information of diagnosis, lesion side, side of vestibular dysfunction, and degree of vestibular deafferentation of each patient with a peripheral vestibular dysfunction (PVD).

	Diagnosis	Lesion side	Side of vestibular dysfunction[1]	Degree of vestibular deafferentation[2]
Patients with bilateral PVD				
1	vestibular neuritis right; vestibular schwannoma left	right & left	right & left	incomplete right; complete left
2	vestibular schwannoma right; idiopathic left	right & left	right & left	complete right; incomplete left
3	idiopathic right; idiopathic left,	right & left	right & left	complete right; complete left
4	endolympathic hydrops right; endolympathic hydrops left	right & left	right & left	complete right; complete left
5	idiopathic right; idiopathic left	right & left	right & left	complete right; incomplete left
6	dehiscence semicircular canals right; dehiscence semicircular canals left	right & left	right & left	complete right; complete left
7	idiopathic right; idiopathic left	right & left	right & left	complete right; complete left
8	idiopathic right; idiopathic left	right & left	right & left	incomplete right; complete left
9	idiopathic right; idiopathic left	right & left	right & left	complete right; complete left
10	vestibular schwannoma right; idiopathic left	right & left	right & left	complete right; complete left
11	vestibular schwannoma right; idiopathic left	right & left	right & left	complete right; incomplete left
12	vestibular neuritis right; idiopathic left	right & left	right & left	complete right; complete left
13	idiopathic right; idiopathic left	right & left	right & left	complete right; complete left
14	idiopathic right; idiopathic left	right & left	right & left	complete right; complete left
15	vestibular neuritis right; vestibular neuritis left	right & left	right & left	incomplete right; incomplete left
16	meningitis right; meningitis left	right & left	right & left	complete right; complete left
17	idiopathic right; idiopathic left	right & left	right & left	incomplete right; incomplete left
18	idiopathic right; idiopathic left	right & left	right & left	complete right; complete left
19	idiopathic right; idiopathic left	right & left	right & left	complete right; complete left

Patients with chronic unilateral PVD				
1	vestibular schwannoma	left	left	complete
2	vestibular schwannoma	left	left	complete
3	vestibular schwannoma	right	right	incomplete
4	vestibular schwannoma	left	left	incomplete
5	vestibular schwannoma	right	right	complete
6	vestibular schwannoma	left	left	incomplete
7	vestibular schwannoma	right	right	complete
8	endolympathic hydrops	right	right	incomplete
9	vestibular schwannoma	left	left	complete
10	vestibular schwannoma	left	left	complete
11	vestibular schwannoma	right	right	incomplete
12	vestibular neuritis	right	right	incomplete
13	zoster oticus	left	left	complete
14	vestibular schwannoma	left	left	complete
15	vestibular schwannoma	right	right	incomplete
16	vestibular schwannoma	left	left	complete
17	vestibular neuritis	right	right	complete
18	vestibular schwannoma	left	left	incomplete
19	Menière’s disease	right	right	incomplete
20	vestibular neurectomy	left	left	complete
21	meningitis	left	left	incomplete

Patients with acute unilateral PVD				
1	vestibular neuritis	left	left	complete
2	vestibular neuritis	right	right	incomplete
3	vestibular neuritis	left	left	incomplete
4	vestibular neuritis	right	right	incomplete
5	vestibular neuritis	left	left	incomplete
6	vestibular neuritis	left	left	incomplete
7	vestibular neuritis	right	right	incomplete
8	vestibular neuritis	right	right	incomplete
9	idiopathic	left	left	incomplete
10	vestibular neuritis	right	right	incomplete
11	vestibular neuritis	right	right	incomplete
12	vestibular neuritis	right	right	incomplete
13	vestibular neuritis	right	right	complete
14	vestibular neuritis	right	right	incomplete
15	vestibular neuritis	right	right	complete

Note. [1]*Side of vestibular dysfunction was categorized according to bithermal caloric testing.*

[2] *Degree of vestibular deafferentation was measured with bithermal caloric testing.*

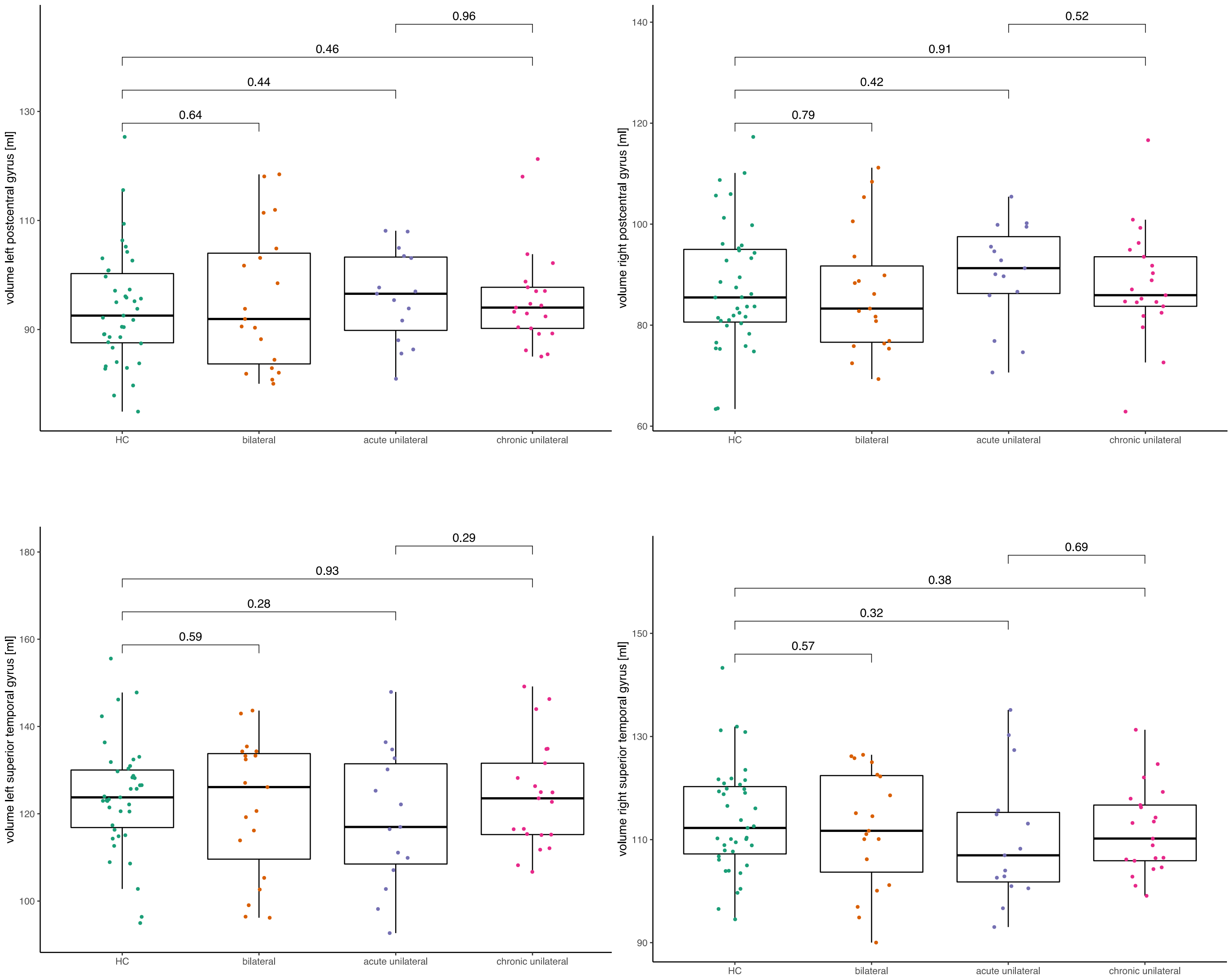

Supplementary Figure S1: Boxplots showing volumes of the left and right postcentral gyrus (first row), and the left and right superior temporal gyrus (second row) in the different groups of patients with peripheral vestibular dysfunction and healthy controls (HC).

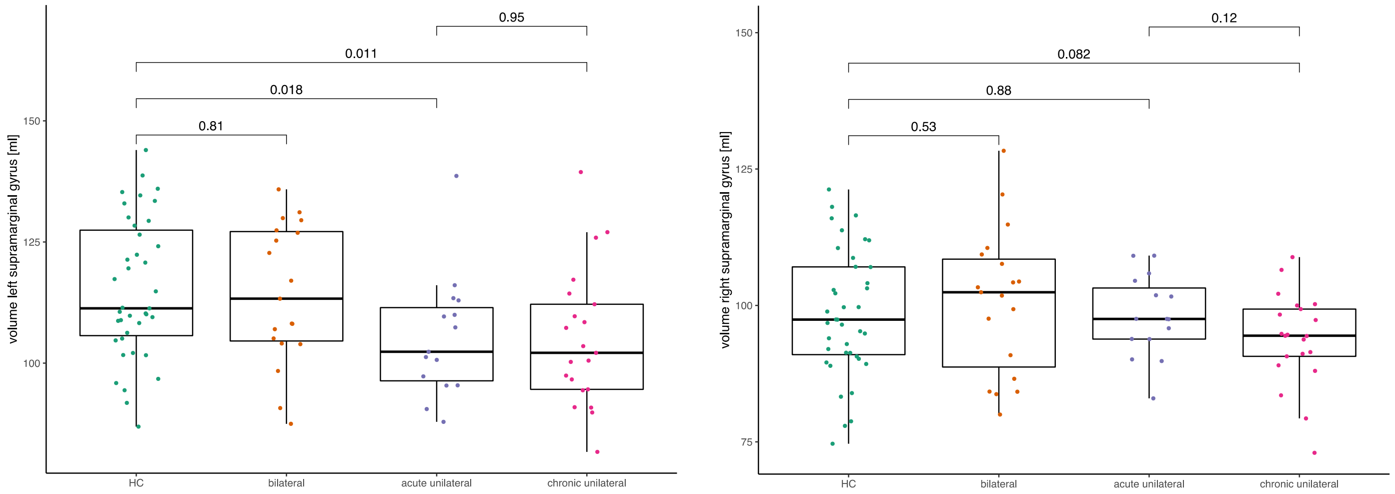

Supplementary Figure S2: Boxplots showing volumes of left and right supramarginal gyrus in the different groups of patients with peripheral vestibular dysfunction and healthy controls (HC).

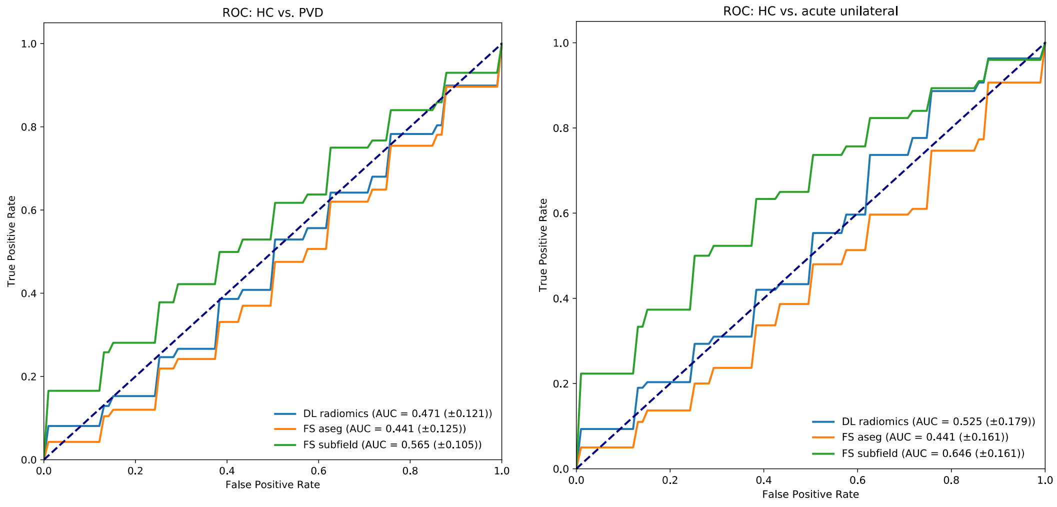

Supplementary Figure S3: Receiver operating characteristic curves (ROC) of the two classifiers for separating healthy controls (HC) from patients with peripheral vestibular dysfunction (PVD, left), and from the subgroup of patients with acute unilateral PVD (right). Shaded areas indicate one standard deviation.

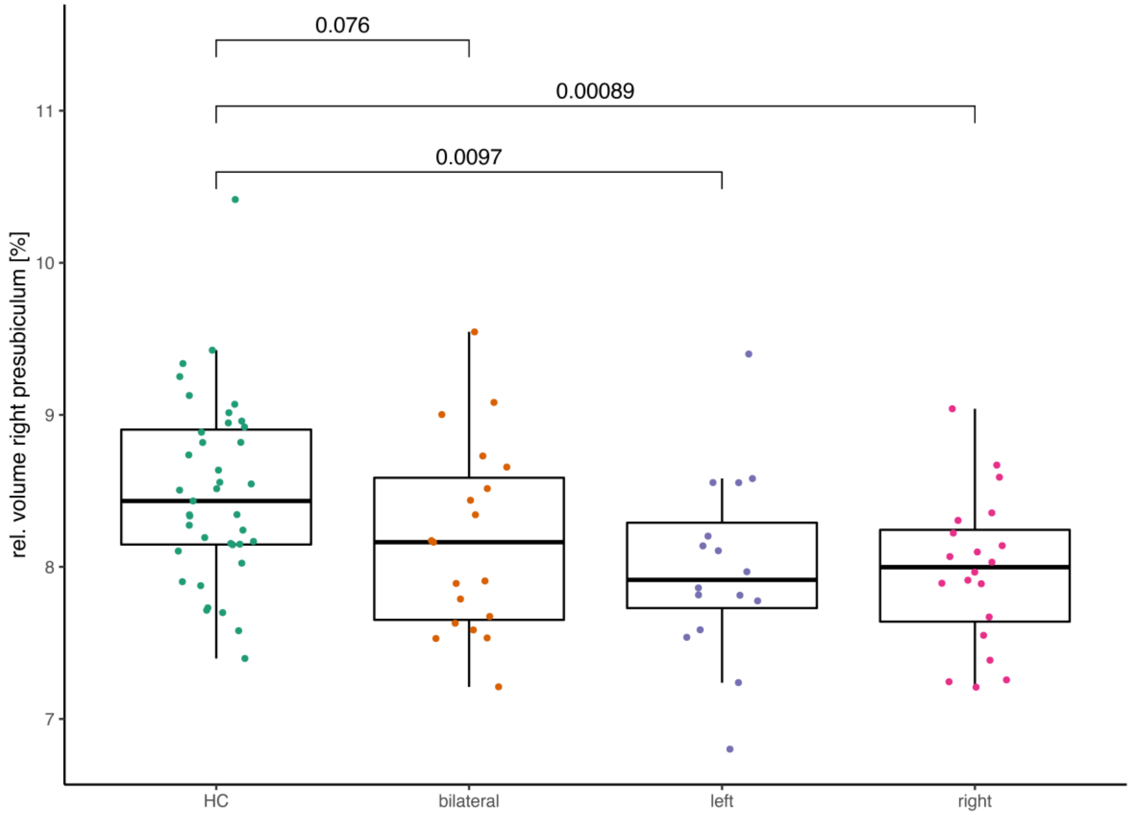

Supplementary Figure S4: Boxplots showing the relative volume of the right presubiculum between healthy controls (HC) and the laterality of the disease of the patients with peripheral vestibular dysfunction.

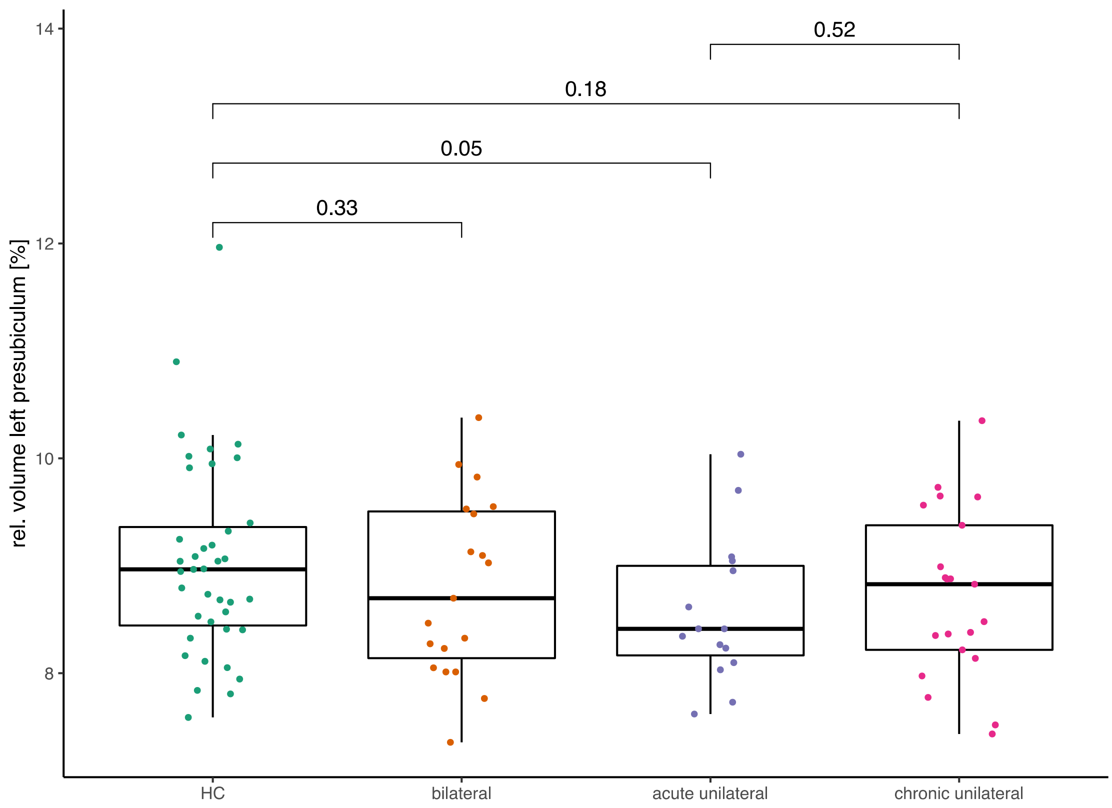

Supplementary Figure S5: Boxplots showing the relative volume of the left presubiculum in the different groups of patients with peripheral vestibular dysfunction and healthy controls (HC).

Supplementary Table S3: Correlation table for right presubiculum and study variables with correlation coefficients (Kendall's Tau), 95% credible intervals and Bayes factors.

Variable	*n*	*M*	*SD*	τ	95% CI (lower)	95% CI (upper)	BF_{10}
1. Presubiculum Right [a]	94	294	38	—	—	—	—
2. Median Response Time Rotation [b]	89	1667	520	-0.103	-0.238	0.038	0.379
3. Response Time Rotation-Control [b]	89	406	224	-0.075	-0.211	0.065	0.237
4. Accuracy Rotation [c]	89	0.95	0.11	0.112	-0.030	0.246	0.452
5. Accuracy Rotation-Control [c]	89	-0.03	0.05	0.056	-0.084	0.192	0.186
6. Color-Word Interference (D-KEFS) [d]	89	10.0	2.3	-0.046	-0.281	-0.001	0.978

[a] in mm^3
[b] in ms
[c] in percent (%)
[d] in age-corrected Scaled Scores

Supplementary Table S4: Correlation table for left supramarginal gyrus and study variables with correlation coefficients (Kendall's Tau), 95% credible intervals and Bayes factors.

Variable	*n*	*M*	*SD*	τ	95% CI (lower)	95% CI (upper)	BF_{10}
1. Supramarginal Gyrus Left [a]	94	11154	1857	—	—	—	—
2. Median Response Time Rotation [b]	89	1667	520	-0.022	-0.159	0.117	0.145
3. Response Time Rotation-Control [b]	89	406	224	-0.134	-0.268	0.008	0.766
4. Accuracy Rotation [c]	89	0.95	0.11	0.133	-0.010	0.266	0.735
5. Accuracy Rotation-Control [c]	89	-0.03	0.05	0.097	-0.044	0.232	0.339

[a] in mm^3
[b] in ms
[c] in percent (%)

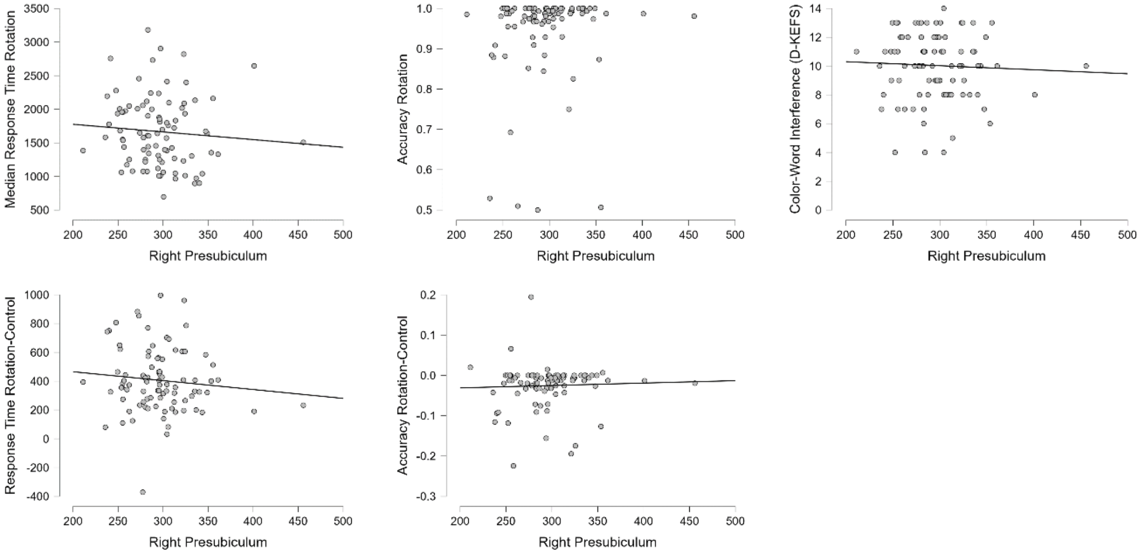

Supplementary Figure S6: Scatter plots for correlations between the right presubiculum and study variables.

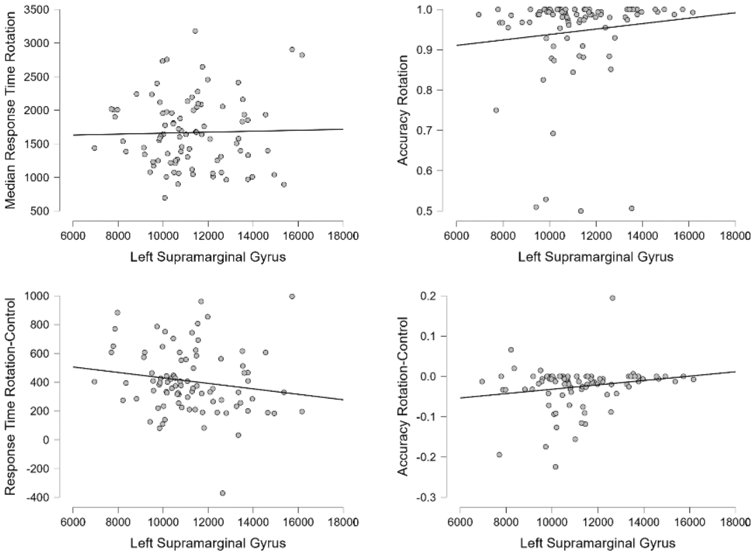

Supplementary Figure S7: Scatter plots for correlations between the left supramarginal gyrus and study variables.
